# Can training interventions in entrepreneurship, beekeeping, and health change the mind-set of vulnerable young adults toward self-employment? A qualitative study from urban Tanzania

**DOI:** 10.1371/journal.pone.0221041

**Published:** 2019-08-22

**Authors:** Masunga K. Iseselo, Idda H. Mosha, Japhet Killewo, Linda Helgesson Sekei, Anne H. Outwater

**Affiliations:** 1 Department of Clinical Nursing, Muhimbili University of Health and Allied Sciences, Dar es Salaam, Tanzania; 2 Department of Behavioural Sciences, Muhimbili University of Health and Allied Sciences, Dar es Salaam, Tanzania; 3 Department of Epidemiology and Biostatistics, Muhimbili University of Health and Allied Sciences, Dar es Salaam, Tanzania; 4 NIRAS Tanzania and consultant, *Ruka Juu na Fema* (television series), Femina HIP network, Dar es Salaam, Tanzania; 5 Department of Community Health Nursing, Muhimbili University of Health and Allied Sciences, Dar es Salaam, Tanzania; Universitat Jaume I, SPAIN

## Abstract

Young adults face unemployment-related challenges, particularly in low- and middle-income countries. Self-employment is encouraged by the Tanzanian government and international institutions such as the World Bank. It has been found that young adults who are employed or self-employed show more functional independence and less inequality and social polarization, as well as a decrease in deviant behaviour. However, limited knowledge and skills related to entrepreneurial activities contribute to lack of motivation towards self-employment among young adults. In order to examine these behaviours, an intervention study implementing an entrepreneurship and beekeeping training in Dar es Salaam, Tanzania was conducted. After completion of the intervention, a qualitative study was conducted that used focus group discussions (FGDs) to explore the experiences and changes in behaviour of young adults following the intervention. A total of 36 of the original 57 young adults from four camps who fully participated in the four arms of interventions were recruited. Qualitative content analysis was used to analyze the FGD data. Three themes emerged from the findings: establishment and maintenance of an entrepreneurial business, changes in behaviour, and perceived challenges. Improved entrepreneurial skills, customer care, and financial management were expressed as positive changes the participants attained relating to business management. Similarly, changes in the participants’ behaviours, attitudes, and lifestyle practices led to improved health and increased recognition and respect in their communities. Insufficient start-up capital and long intervals between sessions were the main challenges. The study showed an improvement in the ability of the participants to generate the human, social, and financial capital prerequisite to business development. Increase in customer care, social capital and financial management are key factors for successful microbusiness activities for stable self-employment.

## Introduction

Tanzania, like many other low-income countries, has a young population, with 70% below the age of 30 years [1]. Because of high rates of unemployment and underemployment, the full potential of young people’s contribution to the country’s economic and social development is not utilized. The national economy is not creating enough jobs to absorb its rapidly growing cohort of young people leading to young adults in Dar es Salaam (DSM) with few employment opportunities. The available occupations for uneducated male young adults, such as day labor, are insecure; formal employment, such as work as a guard or cleaner, often pays less than minimum wage; some occupations, including work in small-scale fisheries and in forestry, are no longer economically viable [2]. As a result, the informal sector has become an important source of employment for young adults. Helgesson [3] found that in Tanzania many of the youth migrating to towns have subsistence farming as a complementary livelihood strategy, alongside self-employment in micro-business. However, the support system for self-employment is still very limited, especially for those without the relevant skills. Farming in young people’s home villages, where their natal families reside, remains an employment possibility. However, it is seen by many young people in Tanzania today as subsistence work, not as something that can add value to their lives [4]. This is true of many youths who have immigrated to urban areas. The Tanzanian Development Vision 2025, an outline for economic and social development drawn up by the national government, requires the citizens to take an entrepreneurial approach toward meeting its development challenges, starting with a change of *mind-set*. For example, schools and institutions should not be educating job seekers; they should educate people with the mind-set of being job creators and promoters [5].

In Tanzania, the problem of unemployment can be reduced if youth engage in entrepreneurship practices as a means of creating employment which in turn will lessen the problem of crime [6]. However, lack of knowledge and skills about how to start and maintain an entrepreneurial enterprise is a problem among young adults. It is reported that educating young adults about entrepreneurship practices increases innovative behaviour, interpersonal skills and motivation for accessing funds necessary for starting a business [7]. Similarly, Olugbola highlighted that there is a positive effect on motivation, opportunity and resource identification among young adults after entrepreneurship training [8]. It is predicted that male young adults who are self-employed show more functional independence, less inequality and social polarization, and decreased participation in deviant acts. This is important for social stability.

Beekeeping is a potential income generating activities for young adults in low income settings. Beekeeping activities has been reported to create job opportunities and increase household incomes]; there is relatively higher income for honey production compared to crop production [9]. In addition to increasing household income, beekeeping offers multiple benefits including nutritional and medicinal products for sale or home use [10] as well as improving pollination services essential for increased crop yields [11]. It is also important to note that entrepreneurship and beekeeping training among young adults helps to increase creativity and innovations towards self-employment [8,12,13]. However, it needs to focus on specific areas where the knowledge gained should be utilized.

In vocational schools, integrated entrepreneurship education play an important role in problem solving, and enables individuals who have received knowledge and skills to plan, start, and run their own businesses [13].

Evidence also shows that, self-employed young adults are less likely to engage in high risk behaviours than those who are daily wage earners or unemployed [14,15]. Ill health prevents a person from fulfilling activities of daily living and increases expenses spent seeking health care services. Likewise, it is postulated that self-employment can be good for mental and physical health outcomes in the society [16]. In this context, training vulnerable young adults on health issues, promotes good health which is a prerequisite for self-employment especially in low income countries.

Many challenges face would-be entrepreneurs among Tanzania’s young adults, including limited education, few employment opportunities, low financial capability, inadequate start-up capital, a lack of entrepreneurship culture and skills, and limited access to information. To address the issues of unemployment, a training program targeting vulnerable young men in DSM was developed and conducted to teach entrepreneurship. This was followed by the present study to assess the extent to which such an intervention could change the mind-set of young men toward the creation of self-employment.

## Materials and methods

### Study design

An exploratory qualitative study was designed to assess the experiences of vulnerable young adults living in DSM while participating in an entrepreneurship and beekeeping training intervention.

### Site and setting

The site of the present study was in Dar es Salaam (DSM), the largest city in Tanzania and a commercial hub for most of East and Central Africa. The most recent population census places DSM’s population at an estimated 4.4 million. Young adults (ages 15–35 years) make up about 46.8% of the total population in Dar es Salaam region, the larger jurisdiction of which the city of DSM is a part [17].

In Tanzania, young men often congregate in camps [18]. These camps had names, fixed memberships of approximately 10–80 people, structured leadership, and specific meeting places; an individual’s membership can last for years. Though not formally registered, camps are informally recognized by local leaders in the city. There are hundreds of camps scattered in all districts of DSM Region. The camps that were the focus of the present study were based in Kinondoni District, the largest of the five districts in the city.

### Study participants

The inclusion criteria for study participants in the camps were that members should be 18–35 years old, unemployed without a reliable source of income, possessing an interest in entrepreneurship and beekeeping. We further selected small camps based on the government’s preferred number of 10–15 members for a viable entrepreneurship group.

### Sample size and Sampling procedure of participants and camps for training

A total of 71 camps were selected. In these camps, six important selection criteria were identified: camp name, number of male and female members, if the presence of a weapon was reported, if the interviewer felt safe, and whether the interviewer was told not to work there. Camps at which a weapon was reported (*n* = 20) or at which previous researchers had been asked not to work for safety reasons (*n* = 5), or where the interviewer did not feel safe (*n* = 9), were not included. Camps that had been chosen for previous research (*n* = 23) were also excluded. That left us with 14 camps from which to select our study population. Among these, 8 were not chosen because members were greater than 15 in number or because the camp had many female members. (Females were not included in this intervention because, compared to males, they were at low risk for receiving or perpetrating violence [19]]. Guided by maps drawn by Yamanis et al. [18], our researchers visited the remaining six camps to discuss the project with the camp leaders. Two of the camps were not selected because their members were not interested in beekeeping. Therefore, four camps remained for the training intervention. Then the researchers introduced themselves to the local government leaders of the areas in which those four camps existed.

**Fig 1:**
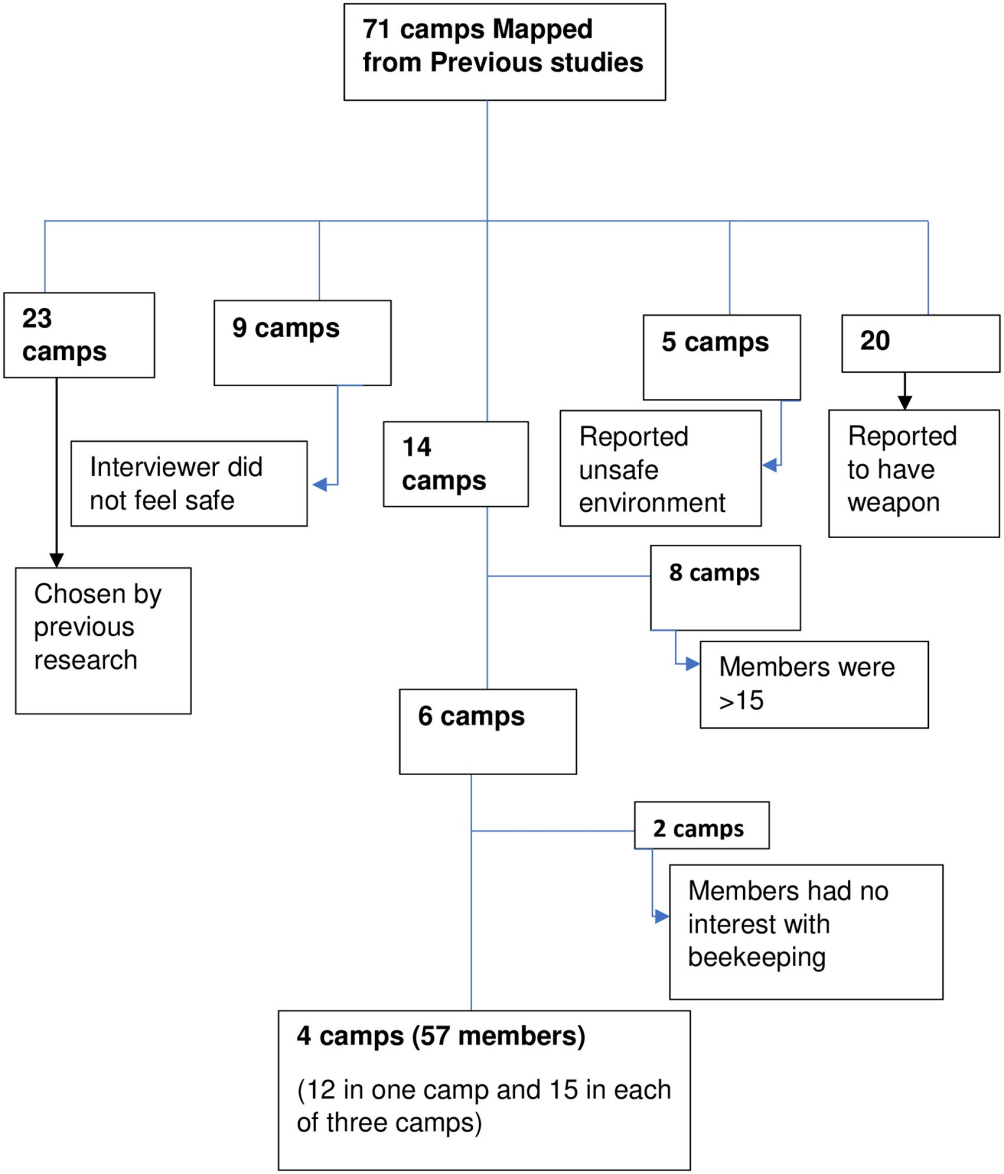
Flow chart for camp and participants selection for training intervention.

Camp 1 had 12 members. Camps 2, 3, and 4 each had 15 members, giving a total of 57 participants at baseline, of whom 2 were female (Fig 1).

### The intervention

There were four arms of the intervention: health only, entrepreneurship and health, beekeeping and health, and all three combined. The 57 young people who attended the first day came from all four camps. The first session was an introduction to the project, and to informed consent. If the potential participants gave informed consent (which they all did), a baseline interview was conducted following which each camp was randomized into one of four arms, as shown in Table 1. These 57 members became a closed cohort that we followed through 10 day-long intervention sessions over 1.25 years, and then interviewed at 3-, 6- and 12-months postintervention.

**Table 1:** Entrepreneurship, Beekeeping, and Health Intervention training sessions, by intervention arm.

No.	Training session	Arm 1 Health	Arm 2 Health + Entrepreneurship	Arm 3 Health + Beekeeping	Arm 4 All Interventions
1	Introduction	√	√	√	√
2	Beginning Beekeeping			√	√
3	Starting Beekeeping			√	√
4	Sources of Capital		√		√
5	Mind Your Health	√	√	√	√
6	Environment, Forests, Bees			√	√
7	Saving, Investing Profit		√		√
8	Business Plan		√		√
9	Harvesting			√	√
10	Marketing		√		√
	Total number of sessions	2	6	6	10

The sessions were based on existing interventions used in Tanzania. The health sessions were based on topics used by United States Peace Corps medical officers training volunteers about how to stay healthy in Tanzania: nutrition, worms, HIV/AIDS/STDs, and first aid. The entrepreneurship training was conducted by the organization behind the *Ruka Juu na Fema* entrepreneurship TV programme; the same videos and trainers used for the TV programme were used for the present project. The entrepreneurship training was oriented toward young people without a lot of education, in order to support them in starting and running microbusinesses. The beekeeping training was conducted by current and retired beekeeping officers of the Ministry of Natural Resources and Tourism. Field sessions took place and beehives were hung in the government forest at Kongowe, 40 kilometers west of DSM.

Different methods were used to train the participants, including lectures, simulations, field activities, and videos. Materials to make beehives were given to the participants at no charge, but none of them received financial capital for any of the interventions.

### Sample size selection for FGDs

Thirty-six (36) of the original 57 members participated in the final FGDs (control: 8/12; entrepreneurship: 7/15; beekeeping: 12/15; all: 9/15). Of those who did not participate, one participant had died as a victim of community violence, three had returned to their home areas outside DSM, and three were employed and were not available for the FGDs. Fifteen others did not appear for the interview for unclear reasons. All participants were males.

### Data collection instrument

A focus group discussion guide (FGD) was used by the moderator to steer the discussions, with the following questions:

What did you like the most about the entrepreneurship, beekeeping, and health project?
What expectations did you have?
Were your expectations met? Did your life change?
How could the project be improved?

Each question was followed by probes in order to explore the issues that arose.

### Data collection

Data were collected in July 2017. FGDs took place as part of the final interviews, 12 months postintervention. Five FGDs were conducted, each involving 7–12 participants. The discussions were conducted by the second author and were audio-recorded. Data collection was done in Swahili, the official language of Tanzania. All study participants spoke Swahili fluently. All FGDs were conducted in a room located on the premises of Muhimbili University of Health and Allied Sciences, in DSM. The moderator (the second author) led the discussions; research assistants took notes and organized the recordings. Each FGD lasted about 40 minutes.

### Data analysis

Qualitative content analysis was used to analyse the data. Analysis began immediately after completion of the FGDs. The second author listened to the audio recordings before transcription. Data were transcribed verbatim. Transcripts were checked by the authors for correctness. Then the authors read the transcripts, line by line, in an attempt to understand the data content (S1 Dataset). All files of transcripts were transferred to NVivo software for coding and organization. The texts were analyzed in the native language of the respondents. We had five transcripts in Microsoft Office Word format. Because the analysis was guided by content analysis principles [20], the transcripts’ files were read thoroughly, and interesting excerpts were coded to free nodes. From these nodes, we created five tree nodes: change in life aspect of young adults after the program, expected benefits from the program, learning health-related issues, important future improvement of the program, and issues that arose during the intervention. Coding of these nodes continued through the rest of the documents. Reflecting the objective of the present study, node classifications were created containing defined attributes for changing the mind-sets of young people toward entrepreneurship as a viable, income-generating opportunity. Memos were also formed for documenting thoughts, doubts, and insights that emerged as we reviewed the data. After we coded all information and organized it into a manageable format, all nodes were shared among the authors for review, and agreement was reached on the coded information. Sharing the nodes helped improve test- retest reliability of the coding system and organization [21]. We continued reading and abstracting the contents to more specific ideas that were mutually exclusive of each other.

This process resulted in refinement of the original tree nodes into the themes and categories. Representative quotes were identified for each theme and category, then were translated into English. The translation of the text was conducted according to Brislin [22].

### Ethical considerations

Ethical approval for the present study was obtained from the institutional review board of Muhimbili University of Health and Allied Sciences. Further permission was sought from the local leaders where the camps were located. Before commencement of data collection, we explained to the participants the nature and benefit of the study and how we would uphold the confidentiality of the information they would provide. All participants agreed to participate and signed the consent forms.

## Findings

### Characteristics of the participants

Most of the study participants were born in DSM. All participants had received at least a primary school education. None had training above the diploma level as shown in Table 2.

**Table 2:** Demographic attributes of participants (*N* = 36).

Characteristic	*n* (%)
**Region of birth**	
Dar es Salaam	30 (83.3)
Outside Dar es Salaam	6 (16.7
**Sex**	
Male	36 (100.0)
Female	0 (0.0)
**Age, years (*M* = 23.0, *SD* = 4.9)**^a^	
< 20	20 (57.2)
20–24	9 (25.7)
25+	7 (17.1)
**Education**	
Primary	12 (33.3)
Secondary	10 (27.8)
Postsecondary training	6 (16.7)
Diploma	8 (22.2)

^a^ Age was not provided by one study participant.

### Themes

Three themes and seven categories emerged, as shown in Table 3: (a) establishment and maintenance of an entrepreneurial business, (b) change in behaviour, and (c) perceived challenges for the intervention. The first theme describes the ability of the participants to initiate and sustain business activity. The second theme describes how the project changed the participants’ behaviours, attitudes, and practices toward overall life aspects. The third theme is related to challenges the participants encountered during the interventions.

**Table 3:** Summary of themes and categories.

No.	Themes	Categories
1	Establishment and maintenance of an entrepreneurial business	a. Increase in entrepreneurial skills
		b. Improved customer care
		c. Improved financial management
2	Change in behaviour	a. Improved health lifestyle
		b. Gaining respect and recognition in the community
3	Perceived challenges for the intervention	a. Long intervals between sessions
		b. Lack of start-up capital

### Establishment and maintenance of an entrepreneurial business

Most participants indicated that as an outcome of the training they had acquired an ability to establish a sustainable business using knowledge gained from the training. Some of the participants reported that they had become more business-focused than before. Others said they had gained experience in customer care that could be utilized if they had enough start-up capital. The experiences and abilities are described in the following categories.

#### Increase in entrepreneurial skills

Several participants reported that the intervention had increased their interest in business issues, though some had already established a group business. They stated that they could do better now since they had gained the knowledge and skills to run a business. One participant said:

*“What I learnt more is that entrepreneurial education is based on how a person can become an entrepreneur*, *and what challenges he can face and address as a business owner*. *And how a person…can come to understand the issue of entrepreneurship*, *how much money one can use in future activities.”*

(Entrepreneurship, Participant 6)

Several participants were proud of the entrepreneurial education they had gained, which could help them to establish their own businesses and be able to employ themselves. One participant said:

*“When I heard there was entrepreneurship education…the day I was taught there*, *I was amazed*! *But thanks to God*, *it will help me to keep myself up-to-date towards self-employment.”*

(Entrepreneurship, Participant 3)

Most participants showed strong interest in initiating beekeeping projects. They said they had gained skills that could enable them to run the projects, except for certain shortcomings as described by one of the participants:

*“I look forward to beekeeping*.*… I expect to be a good beekeeper*. *But how will I be a good beekeeper*? *I will have to get to the end*, *I’ll know*. *At one point you have got the beekeeper education*, *and you’ve understood everything [about] how bees are kept*, *everything you can do*, *but you do not have a plot of land* [where beehives can be placed].”

(All interventions, Participant 2)

#### Improved customer care

Participants reported increased knowledge and skills in the area of customer care. They said they would use their improved skills when serving their clients. They all admitted that they had had poor knowledge of how to deal appropriately with clients before the training, as the following testimony illustrates:

*“I thank Fema facilitators for entrepreneurial issues*, *about how to talk to your clients*. *There was a video screen displayed at the training*. *You see this guy*, *whom you should not be like*. *Because when the client comes*, *he spoke in an unfriendly way*. *However*, *there is someone else who is civilized*, *and welcoming customers well.”*

(All interventions, Participant 5)

Another participant said that:

*“After getting this kind of education*, *I usually tell my relatives at home that the customer does not have to be harassed*. *You have to act like a civilized person*. *Have a smile*. *A customer loves you*, *but if you’re angry all the time*, *really the customers will run away.”*

(All interventions, Participant 3)

#### Improved financial management

Almost all the participants were engaged in some sort of entrepreneurial activity, and most said they had gained an increased ability to manage their business, especially in regard to budgeting and controlling net gain. All money obtained from any income source was designated for specific uses, as one participant explained:

*“If you work*, *install something; if you want your stuff to go in a certain direction*, *your budget has to be precise*. *For my part*, *it might have changed*. *For example*, *this money is for the bus fare*, *I’ll spend it only on fares*. *There’s just a certain order I’ve got*, *on my budget.”*

(All interventions, Participant 8)

Others expressed concern about the extravagant spending that consumed much of their earnings. Avoiding costly things, like soda drinks, in the present could help them save money for the future. One participant admitted that:

*“We pretend we are ignorant about how an extravagant life now can ruin our future*. *Luxurious things like entertainment have always existed; before we were born*, *the luxurious life was present*. *People die*, *but the luxuries still continue*. *So*, *you first have to avoid extravagance and do beneficial things that are in line with a good life*, *so that you can get anything you want in your future”*.

(All interventions, Participant 4)

### Change in behaviour

Most participants reported that their standard of living was better than when the program started. They reported having changed in terms of financial management, living a healthier lifestyle, and gaining respect and recognition from the community.

#### Improved health lifestyle

Most participants noted that they had achieved a healthier lifestyle that included improvement in what they ate and improved knowledge about preventing illness and providing first aid to others. They reported that before the program, they had health practices that resulted in poor health, and thus spent more on treating diseases. As one participant explained,

*“So*, *you can give me an entrepreneurial education*, *and I can succeed to have a hundred million shillings*, *but if I do not know the principles of disease prevention and I am drinking unsafe water*, *I will get stomach problems*. *Then even my entrepreneurship or programs will collapse because I am sick.”*

(Entrepreneurship, participant 4)

Others stated that they had changed from being promiscuous to conducting themselves in a more sexually responsible manner. The following comment is characteristic of this change:

*“All my friends knew me as a very promiscuous person*. *The sexual education I received has seriously changed my sexual life*. *A condom to me was like…*., *“Ah*, *you cannot eat a banana with its outer coverings”* [i.e., a man cannot have sex using a condom].… *You know our street words*, *eh*? *I’m thankful I’m no longer like that*. *My character right now is understandable.”*

(All interventions, Participant 7)

A member of the control intervention arm added,

*“In short*, *this project has been the most successful activator for the life we live in the street from sanitation to pursuit of money*. *Given the life we lived before joining this project*, *it was a very difficult task when it came to eating*: *You do not wash your hands*, *because you are eating with a spoon*. *So*, *there was always a fever in the intestines*. *But after having a little health education*, *I now know that even though you eat with a spoon*, *you must wash your hands again with soap.”*

(Health, participant A7)

#### Gaining respect and recognition in the community

Most participants appreciated that the training changed the way they were perceived by community members. Previously they had been counted as useless people who could not contribute anything to the family or the community. After the training, participants said they had gained respect and recognition. These statements are representative:

*“My life has changed in many ways*. *You know*, *you attend this seminar every day*. *You learn to talk to older people*. *You are learning to talk to respectable people*. *I did not have the initiative to speak in a dignified way to people or even to be respectful!”*

(All interventions, participant 9)

*“You know*, *sometimes when we returned home from the entrepreneurship training*, *you’re asked*, *“Where are you coming from*?*”*.*… We used to answer*, *“We are coming from the college*, *to do something good*, *with someone* [the researchers]. *For the people around us*, *there’s some respect right now they give us.”*

(All interventions, participant 8)

Others felt respected and honoured to be followed to their own residences by the program facilitators, something that might have made them feel ashamed because they felt that their neighbourhoods were too slum-like for the facilitators to visit. This increased the respect shown to the participants in the community:

*“We think about the environment we came from; we’re from the slum*, *maybe I can tell you…*. *The first day when Dr*. *Anne* [the researcher] *came*, *wondering just what the environment was*, *we felt (we) from the uswazi* [slum areas] *and her were two different things*! *However*, *what we expected was not how it was*. *We were considered; for example*, [during training sessions] *at the time of eating*, *we were given good food to eat*. *So we liked it*, *we liked it generally.”*

(Entrepreneurship, participant 7).

### Perceived challenges for the intervention

The participants indicated that there were several challenges that were obstacles to initiating entrepreneurial activities. The participants explained that it was difficult to retain what they had learned when the intervals between sessions were long. Insufficient start-up capital was a major concern described by many participants. A lack of accessible places (bush or forestland) to situate the beehives for those who showed interest in beekeeping was also a major stumbling block. The forests in which the hives had been hung were too far for affordable or timely access.

#### Long intervals between sessions

We designed the project in such a way that participants were trained at different intervals, according to the intervention arms. There were two major delays in completing the training sessions: (a) a halt in training because of the national presidential election campaign of 2015 and (b) a long delay before bees moved into the hives we had hung, which caused a six-month postponement of the final session (harvesting). All intervention training was completed in 1.25 years.

Most participants expressed concern at the great intervals between training sessions. The respondents of the Health arm intervention, for example, were first interviewed in April 2015, received the health session in June 2015, then were called for the second interview in October 2016. Participants said that the intervals between sessions were so long that some of them had difficulty remembering what they had learned in the previous session. As one participant observed,

*“Its process should not be long like this*. *For when someone studies something—the first topic was the first month*, *and then the second session is in the twelfth month—it is most likely a person can forget what he studied in the middle*. *Because someone’s head sometimes has many things in it*, *and many of us have aged and have many responsibilities”*

(Entrepreneurship, participant 6)

Another participant stated,

*“On my side*, *they should not have done the project for such a long time*, *or if it was a long time*, *it would not take much time to give someone some knowledge of something*. *As we used to spend three months*, *four*. *Here now* [due to delayed intervention sessions], *it reached a time we stayed up to seven months*. *I see that people will fail to understand things quickly”*.

(All interventions, participant 8)

Some participants suggested alternative schedules that could help them understand the training material better. A member of the Health arm also expressed the opinion that more than one intervention session would be a good idea:

*“If so…we might be doing twice a week*, *or every month four days*, *one month comes four days*. *For example*, *every Saturday you are attending*. *It would give us a little more knowledge”*.

(Health, participant A3)

The long intervals between training sessions were not optimal for learning. However, an unintended consequence was that many participants demonstrated that they were willing to stay engaged with such an intervention for longer than 2 years, even with very little formal input.

#### Lack of start-up capital

Even though the participants were told several times that they would receive no capital, it was a topic of ongoing discussion. It seems that most participants expected that they would be given capital to start a business after completion of the training. A member of the beekeeping arm explained:

*“They should help us financially*, *as well as find us places for hanging the hives*, *because even the hives cannot be found free of charge*. *Even farm owners cannot accept us [to hang our hives on his farm] if we don’t have money*. *For this reason*, *farms are hired*. *I feel that it is better to have fees for the field and other facilities which are involved in such issues”*.

(beekeeping, participant 1)

Another participant from the entrepreneurship arm said,

“*We have gained entrepreneurship education*. *But entrepreneurship teaches that business does not start if we have no money*. *We have education*, *but we don’t have the plot* [of land to hang our hives]. *So*, *we ask if there is opportunity to be enabled* [to get start-up capital]; *then our hope is to implement what we have learned now and to work”*.

(Entrepreneurship, participant 2)

A similar expectation of start-up capital was expressed by another participant:

*“We have learned all things*! *At the end of the day*, *what will the end be*? *Everyone here awaits the end of the day*, *how does the picture end*? *Everybody is waiting*, *what will we finish with*? *Yes*, *I have learned entrepreneurship*, *health*, *cleanliness of the environment*, *beekeeping*. *Everybody is waiting for that part* [start-up capital]”.

(All interventions, participant 1)

Another participant had a different perspective on how they could find start-up capital: They knew exactly what was needed, and the knowledge they had obtained could enable them to get it:

*“Knowing that we are coming here*, *many people have paid attention to bees because the main topic is bees*.*… We have been told this is an empowerment project*. *As it is to empower*, *in that sense*, *you will look at your plan*, *what you feel can bring you money*, *that if you make it happen*, *you will get it*. *So*, *you work through it*.

(All interventions, participant 8)

In summary, many participants stated that their well-being had improved as a result of these small training interventions. Many reported that they had gained the skills to start and maintain a microbusiness. They reported personally having changed in terms of more effective financial management and a healthier lifestyle, as well as gaining respect and recognition from their own communities. Many participants were interested in initiating beekeeping projects, but the government forest was practically inaccessible, due to cost and time constraints. Other challenges were overly long intervals between training sessions and a lack of start-up capital.

## Discussion

The present study explored training interventions in entrepreneurship and/or beekeeping as ways of engaging vulnerable young adults in productive activities. Changes in behaviour that enabled the development of important skills for initiation and maintenance of entrepreneurship activities were one of the most important findings of the study. This study has highlighted some changes that were attributed to these few training sessions: ability to establish and maintain an entrepreneurial business, healthier lifestyle, and greater respect and recognition in the community.

The present study revealed that the skills needed to establish a business increased among many of the participants after the training interventions. Similar findings have been reported in other countries including Nigeria, Sri Lanka and USA [8,23,24]. However, the findings might not reflect the reality in Tanzania due to difference in economic level of these countries. Furthermore, our study revealed that human, social and financial capital are important aspects for initiating and maintaining entrepreneurial activities. This is also elucidated by Fatoki that, to develop and maintain small and medium-sized enterprises (SMEs) depends on human, social, and financial capital [25]. Even microbusinesses depend on these three types of capital. The categories that arose from the participants around the theme of establishment and maintenance of an entrepreneurial business were factors that contribute to a successful business. Our findings also indicate that if these factors are utilized effectively, vulnerable young adults can establish their own business as a basis for self-employment. In the United State, for example, individuals with low human capital, wealth and entrepreneurial entry were negatively affected when attempting self-employment [26]. In our interventions, many respondents reported having gained enough knowledge, skills, and interest to run microbusinesses that would lead to successful self-employment.

We have observed that financial management is an important factor for maintaining an entrepreneurial business. This is similarly revealed in various studies on which improvement in financial managements contributed significantly to entrepreneurial skills [27] and was affected by income level, educational level and workplace activity [28]. In this case, our studies aimed to improve financial literacy and management so that young adults can live independently through establishment of small enterprises such as part of self-employment. In this context, financial capital includes equity capital and debt. In Ghana, it was reported that financial capital is needed for a new business, but debt can negatively affect SMEs [29]. In the present study, improved financial capital was most commonly expressed as an increased ability to manage a budget. The participants’ self-perceived increase in fundamental financial management skills indicates that many young adults currently lack skills in keeping and managing money for maintaining a business. The training intervention has substantially improved their budgetary management necessary for maintaining a given business.

The customer care training in the present study seems to have inspired our participants to a notable change in entrepreneurial behaviour. In this instance, improvement in customer care implies that young adults can be successfully trained and be transformed to practice entrepreneurially oriented behaviour. Customer care is associated with social trust. In the USA, individuals in communities with high levels of social trust were more likely to be self-employed compared to individuals in communities with lower levels of social trust [30]. This is also reported in England where customer care was integral component of human relation competencies [31]. However, the findings in these countries might not be comparable to our findings due to economic differences as Tanzania is designated as a low-income country.

Behavioral change as reported in our study is not only good for individuals towards developing healthy lifestyles which are important for entrepreneurship [32], but also important for personal growth and recognition in the community as part of social capital [30,33]. In terms of health intervention, we did not expect the health session to be as important as it appears to have been. Changes in behaviour with respect to a healthy lifestyle were reported by the respondents as a result of a one-day health session. The participants suggested that living a healthy life could lead to increased productivity and that health-seeking behaviour could result in fewer ailments. Similar to other studies, social support from parents is associated with higher levels of healthy behaviours across adolescence and adulthood [34,35]. This implies that multiple behavioural and disease conditions often coexist in the same individual, resulting in a cumulative risk of poor health if intervention is not provided. Training of young adults on health-related matters can help to improve their health, as we found in our study. In the absence of such training, Mukherjee observed that if an individual becomes unhealthy and falls sick, then that person becomes unable to work for economic gain, which in turn makes them poor and sick again [36]. Health and self-employment have a mutual relationship. In the Netherlands and USA, self-employment has been associated with good health outcome [14–16] which is a key factor for successful entrepreneurs.

Recognition in the community is a reflection of community perception about young adults who are vulnerable due to poor parenting and/or poor adult behaviour [37]. Entrepreneurship and beekeeping training intervention facilitated recognition of these vulnerable young adults as potential people who can work productively to help themselves and their families. Recognition is an important aspect of social capital that supports initiation and maintenance of entrepreneurship activities. Previous studies in DSM have revealed that community violence is associated with unemployment and underemployment, which resulted in the resort to theft to meet basic needs of family and individual survival [2,19,38]. Our project has improved this perception significantly and we witnessed a willingness at the government level to allow beekeepers to place their hives in government forests. In other countries, studies have revealed that intervention related to improving social capital promotes entrepreneurship behaviour towards self-employment [32,39,40]. It is important to note that participants’ recognition and respect in the community, can be attributed to a perceived decrease in violent and rude behaviour that is a result of entrepreneurship and beekeeping training. Therefore, it can be understood that the intervention training might have changed some of the attributes of the participants that were perceived as threatening to the community and therefore can undertake productive microbusiness through the regained human capital.

The important perceived challenges reported by participants were strategic or methodological in origin. Start-up capital for initiating and maintaining a project is one of the strategic challenges highlighted in our study. Similar findings reported in Belgium, where it has been found that entrepreneurs often did not know about the available finance alternatives which was the barrier for initiating a business [41]. In spite of capital problems, there are other solutions available. For example, it is possible that young adults in Tanzania can obtain the necessary capital to initiate and maintain a planned entrepreneurial activity through existing institutions such as village community banks (VICOBA) and savings and credit cooperatives (SACCOS), with some guidance or training. Supporting the above statement, Marta et al. in Tanzania reported that loans from Microfinance institutions (MFIs), SACCOS, and VICOBA are available and the most accessible external capital by women entrepreneurs, followed by government subsidies and informal capital such as loans from friends, relative and investors [42]. It is therefore suggested that training to enable young entrepreneurs to access these options be included in future entrepreneurship interventions.

The most important limitations include the following; Firstly, there were no interaction boundaries between the participants before, during, or after the intervention. Although we selected camps from different localities, the members all came to a central location to learn. The respondents did extensive networking and information sharing during and between sessions, making it difficult to qualitatively evaluate the effect of each intervention separately. However, during the FGDs, participants were instructed to relate their own experiences as they participated in their respective intervention arms.

Secondly, only males were included in the current intervention training. It is well known that there is a gender difference in issues related to financial management that might affect the participants in developing entrepreneurial skills especially married women in African setting [43,44]. In our study, there were only two females who, however, dropped out from the training just after the first two sessions. Therefore, gender aspect was not possible to be addressed during the intervention. We recommend that when planning future training intervention on entrepreneurship and beekeeping should focus on females as young entrepreneurs.

Another limitation is that, these findings are based on a small sample which was limited to 36 out 57 participants. This provides a potential lack of data saturation as there were no additional participants who could add new information during data collection. Nevertheless, the authors elicited as much information as possible from the participants. This helped to get the relevant data that make these findings valuable for the entrepreneurs and beekeeping activities.

Lastly, it is not clear what aspects of the program have led to apparent success. Some research suggests that the special attention and extra support such programs provided for youth can lead to positive outcomes (i.e., a Hawthorne effect) regardless of the specific design features of a program [45,46].

We recommend that these preliminary qualitative results be informed by quantitative measurements that could inform researchers about the effect size of the interventions, including whether the participants’ incomes changed over time and whether cases of violence decrease.

## Conclusion

The present study has documented that after a training intervention on entrepreneurship, beekeeping, and health, for underemployed young men led to increases in customer care, social capital and financial management which are the key factors to building, managing, and sustaining microbusinesses necessary for self-employment.

## Supporting information

S1 Dataset This is the raw data and abstracted contents that formed categories and themes. (PDF)
