# Supplementary material for: Can training interventions in entrepreneurship, beekeeping, and health change the mind-set of vulnerable young adults toward self-employment? A qualitative study from urban Tanzania
Source: PLoS One. 2019 Aug 22;14(8):e0221041. doi: 10.1371/journal.pone.0221041 (PMC6705837; doi:10.1371/journal.pone.0221041)
Supplement: S1 Dataset — (PDF) [file pone.0221041.s001.pdf]

SI: Raw data and abstracted contents that formed categories and themes

Change in life aspect of vulnerable young adults after the program

Financial management

Entrepreneurship 1： For my part I am aware of some things, like entrepreneurship. Doing business, how to invest your business, how do you get it? In food, clothing and other problems. So also, in the wild, on my part is something that has changed me.

Entrepreneurship 7: I am a businessman. I see a big change that I have seen in my business, I have been taught how to entrepreneurship. In the beginning where I was there, I did not get a business of entrepreneurship, I was doing business, not looking at income, profit I did. I knew I was using it - working, tomorrow I will find another. But after coming here, after receiving the lessons, that we must respect what income you are looking for. Because you get tomorrow or missing. I am very grateful; I have changed so much with respect to my income in my work where I get money.

All intervention 8: If you work and keep the amount, you don't know anything you have to have your things going this way and so, your budget has to be this way and so forth. This across the fare, I will use on the fare. this across the eating, this is probably the voucher or of what. That is the only order that I know, of my budgeting

All intervention 4:……, so we are ridiculous because of the enjoyment. So now there is nothing else and nothing else, I find it setting. Comfortable, comfortable things exist, because we have since been born happy. Or people die, still enjoy it. So, ah, you see you do the best things that are good for the good life, so that you can get anything you get. So, I learned one good thing. My expectations now that I have found here, I know you here, or you have to have your good budget. So, I have come to learn it. So even when I work, I only know my expectations I put it.

Entrepreneurship 6: For me after coming here and learning this beekeeping project, something that first changed me, when I got the first money; first I think how much I can isolate you for the future. And how much it is in emergency matters. How much would I spend on food and other

things? For that before I also used it, I could think of how my money was spent until I was able to withstand the whole month. That's what I learned.

Improvement in healthy lifestyle

Beekeeping 5: Ah, for me here, it's… ..., my life is to say, that is a little bit changed, though not so much. But I think the future can change as we go. But right now, how I have benefited or changed, that, in the case of…. I have received education on some of these nutrition issues. That I was for instance if I was eating unhealthy foods simply. Now at the end of the day I find myself eating and I am also a change. Now the end of the day come to get here education - of about the dietary issues, the balanced diet, this and so on. My body is that there are foods that I have learned that if you eat this food and this and that, there is a body to build up, there is something to strengthen the body. So that is, my body and now I see little has changed, unlike the time I had no knowledge of.

Health B 6: If you talk about perhaps in these worms, drinking hot water, filtering water, I did not know, I was just picking on the tap, drinking. I've got an education, and so far when I got that I can sit with other people and advise them, sir in a particular project, we've read something and something, that you didn't know, I could tell you when you understood.

Entrepreneurship 4: Because I know that ok, so you'll have to be within a week, I have to eat protein, and that's this food. Ok, even the community I had, I know quite ok, in this week I don't eat protein, I don't eat vitamin, I don't eat it.

I will come back to another ok, I must go to bed is important, I will get malaria.

Drinking water will be fresh to boil. Not to me and to the community, that I will be like a pre-educator to my community.

So you can give me entrepreneurial education, I have a hundred million, but I do not know that the water I used is not clean.

All intervention 7: All my friends know me that I am a prostitute. Yes, but there are sex education, here and what seriously comes. The condom to me is like …., you can't eat bananas and pods, … our street words eh. But I am grateful, I am here, my character right now is understandable.

Bosnia 5: Yes, this has helped that even in a village where there are no health facilities, then I can give first aid, to someone who is suffering from stomach, or long-term vomiting.

Respect in the community

All intervention 9: My life has changed a lot. You know, meeting these seminars every day, you learn to talk to older people. You learn to talk to people of respect. I had no plan to speak respectfully to the people.

All intervention 8: First as a group, there are some places I feel we have moved. Know there is one thing, there is a place we all come out together. When you come from where we are - we come from white, to do something, and something fulan. For those people around us, there is a certain respect that they come to us. That is, the whole group, ie, leaving the individual, changing certain things, but as a group they see these have something that they do, that most people think they have something they do.

All intervention 9: Enhee, from the style we heard, seeing someone does something extraordinary, it's normal. Now for us, a man of wisdom, is a wonderful person to us. Ah, is this wise man today? Or, these children have become white!! For life I have changed life style, such seminars make me now even at family meetings where I was not heard, called now, because I have become a man of wisdom. Now known, this man out, has gone to a place that is known to Muhimbili.

Also I feel free, I had no dream of entering to the college. I had no dream of entering even such a office, staying like this, discussing with the intellectual people like you. I have changed, there are things that have changed.

All intervention 9: …… we started the truth. Then this project has given us respect in the street. You know we all come out, we come from the whole tabernacle. We leave the morning, where are you going? You tell us we are going to Muhimbili. ie you say to the Muhimbili, we go to Muhimbili College, someone I don't know…. we have a short respect. This project gives us respect in the street.

Minor or insignificant changes

Beekeeping 2: Yes. The same is true, because they have not yet given us a particular area, or maybe money, so that we can keep up with our project. But if they would give us a place or money the I would do something, I would see a change.

Notes B 3: In fact, life has not changed, the same life. Why are you told, each goat eats in the length of his string. So we started from the beginning here, for us as we have said, Here I would like to talk to you perhaps with all my friends. Nothing we found.

Health B 2: For me in my life you see right now changed, that has changed but the same

Step 9: I like myself, my life I see has changed a bit. Because I didn't know the manufacture of tanks and beekeeping.

Living independently

Health A 3: I get a little livelihood. Formerly I was… What they found I was looking for. Now I am not dependent on someone, independent. Yeah, what shall I answer you?

Health A 7: Depending on the parent for everything. But right now, I can do anything that is under me, I can do it. …… Eating, clothing and shelter, all I can afford

Expected benefits from the program

Bee-keeping Education and Experience

Entrepreneurship 1: I knew that we would only be taught in class and then you should go to the streets to do your work. But for my part I am very comforted, especially when I got to Rukwa, I learned a lot. Because we went into the wild, we hanged the tanks, first before, we went into the class and learned how to make tanks, Tasnuka, the bee smoke to be no contact, be gentle, I didn't know

Entrepreneurship 6: If I say the expectation, I am talking about getting the knowledge of beekeeping in practice. Because that was the most important thing. When he goes (Rukwa) one person will represent in a group, the education that he will find to educate his friends there is a certain difference with a person going two or three people. There is a difference because one goes to this man if he has missed some items, there is no extra person to help him that is where

the person has missed. So if they had gone even if two people, or three, or possibly a small group would help us,

All intervention 2: I am looking for beekeeping. So far, I expect to be a good beekeeper. Say exactly how can I be a good beekeeper? We have to come to the end where we know, another time you have read the beekeeping education, and you have understood everything that bees are biting this, everything you do, but the field is not. But if it comes to the facilitator, he lets you become a good beekeeper.

Beekeeping 12: We can also find the bee brooding equipment, or equipment to go to the honeycomb.

All intervention 8: …… .. Beekeeping is one of the most difficult issues for young people right now. learning the truth we have learned, but without advanced management, add. Tanks make everyone knows. We don't even see it. This field, wood is here to cut themselves. I don't know everything to do for ourselves, no one will fail to make a hive, and put it on honey people get it. But now that you come, looking at the whole process, If you look at the one who wants to beekeeping, he will charge you for everything. But at the end of the day you give him all, if you give him this bee, without any management, no one will keep the bees. Or if he will keep the bees, I don't think he will be careful for the whole year

.

Lack of Capital

All intervention 1: Yes we have learned all things! At the end of the day, what will be the end? Everyone here waiting for the end of the day, how will the picture end? Everyone is waiting, what will we finish? Yes I have learned entrepreneurship, health, I don't know the environment, beekeeping. We will leave this way: That is, everyone is waiting for that section which… ..

Health A 2: What I want to say, is just asking for help for what we wanted to do to help us get far. For we have given ourselves to school to ask for a location. So what makes us so small is to build up the shelves, and get a little capital to develop it.

Beekeeping 5: maybe we finish this first section, maybe the second will enable us now for this thing we have learned. That maybe they can search for a place or scare us, so we can do this something we have learned. Because if we have learned something then the end of the day we are going to stay without any benefit, knowledge will be lost.

All intervention 8: Find out if we are coming here, many people are looking for bees, because the main theme is bee…. we have been told this is to enable. if it is to enable it you will look at your plan. What can give me a whole, what you feel, this I do, I will crush, the same you go with.

Entrepreneurship 2: We have found a bee education, we have learned entrepreneurial education. But entrepreneurship is that the business we have not started due to lack of money. Beekeeping, we have not started it because we have no location. We have education, but the area we do not have. So I ask if there is capacity to be enabled, then our expectation is to implement that which we have learned now to do.

Beekeeping 1: Empower them financially, because even the tanks themselves cannot be found free. Yes, there are timber there. They even have a farm that can not even accept it. we can pay for the farm fees, and some other items, which are associated with these issues.

Health A 7: Because the shop will depend on, I think you know yourself, frame as another frame tells you about six months. yet, in my mind — that is, in my mind — if I find it, that is, if I am enabled as a whole, it will be enough, ie I am ready to open that business, ie eggs

Attitudes towards the projects

All intervention 5: We others, we are many, but others probably so they eat to eat with that thirteen thousand. But I eat with the thirteen thousand I take, but I also rely on, not to leave me. Even if they don't hire me, even if they look at me and basically nothing to do. Mie my difficult situation, work I want.

Kitankipesa 8: Now on our part we employment is an obstacle. Then many people in our hearts believe that, who this government, there are many things to do, even if the bee hits fail, look at the other side, the possibility of getting a job

Health 3: Because I prefer to go there, Kibaha there, that I am going to have to follow those insects. That is, I love livestock.

Met and Unmet goals and expectations

Health A 7: After meeting with others, we learned many things. But in learning, we have not finished. We are still in training, so I believe, when we finish we will find something that we would never find if we were on the street. So we will work for something that we will get, and we believe we will develop it.

Health B 3: We have been trained in education. But it's not that training here, say that you see it like this… ..But the education we've got.

Entrepreneurship 5: We hope we will be given education, at the end of the day we will go to the scene, see how the beekeeping, and its general preparation. But now I've just got a class education, just know to skip over with bees, and just give the seminar. But now I don't know how to hibernate? how are you made? How are you? So my expectation that is intended for me to come Here I come up with the bees I did not reach.

Health B 6: Because, we have been given bee education. And we agreed, that if there were places, we would be ready to do the bee's business. And then we become the biggest honey seller in the city.

Beekeeping 1: But my great expectation, if here they continue to teach us more. If you look forward to getting income, I will be able to start a chicken egg project.

Health A 3: Now we had a plan to learn bees, because they are teaching us. Now our goals we knew, or my goals, to get far away… I have a honeycomb… a bee farm.

Health B 6: Yeah, of this project. Because we planned, we talked that you are ready for doing that, and we are ready, yes. We are coming every Sunday, every Sunday. , it came to an absolute silence, came to silence, no longer, again, as today we are called for more interviews.

Health A 2: My goals to join this capital (project) is to be the largest man in Tanzania. The first selling honey. I am a very popular person. Finally let me open maybe my branch to sell honey, and that's why we're here to get education. To give us enough education in entrepreneurship, we must continue.

Health A 3: Enhee. Because you have nothing, you see the Lord, what you say to make without planning, you see the master. let me know what I sell the clothes, you see the master, so many clothes.

Establishing and maintaining entrepreneurial business

Health A 7: I see in the forefront of success, my future goals, to become an entrepreneur who can self-employ. I did my work, and I was able to hire my colleagues, and they knew they could earn income. So my future aspirations can be self-employed, being a nimeimprove in beekeeping

Health A 3: There is a chicken, we grow. And so we want us, even if we say, sir, make us a plan and we will see you. The chickens every week we buy a puddle, we take it there. We started with

five, and in the other five they gave, and now we have reached them. So far we have fourteen chickens, (14)

Health A 5: There are many things that we have learned here. What we ourselves could have started in the street… It gives us a morale, motivation. Even if he says, 'Come, let me see!' You show this here.

M Health A 3: We as a group, we have our project we want to introduce. We would like to support the support, there is a project for raising chickens, there is a project for beekeeping.

Health B 1: We would get two car wash machines. In our village there are people, let's say in a village registered a motorcycle as twenty. Twenty motorcycles, every one motorcycle is rolled into two thousand. So for a week it might be just one hundred thousand of us to say. Here we could, after six months, we could buy a pressure pump.

Health B 7: For that store will depend, I think you know yourself, frame as another frame tells you about six months somewhat. Still you have not taken a load, as you say I take maybe the egg tray, how many tray I take, and how many shillings. But in my mind, if I am enabled as a whole, it will be enough, I am ready to open that business, of eggs.

Entrepreneurship 3 But that when I heard there was entrepreneurial education that day we were taught there, until I was surprised! But what I thank God for finding me will help me to develop myself, self-employment

Entrepreneurship 6: What I have learned More is the entrepreneurial education, from how one can become a entrepreneur, and the challenges that the entrepreneur faces.

And what else I learned that, how can someone… how to get into the business of entrepreneurship, how a person can budget his money in future, future.

Health A 1: So much that even when I open a business, even if someone opens the business I can guide it well

Customer care

All intervention 5: I am very grateful to the people of feminine. These entrepreneurial issues, talk to your client. Because there was a screen at all, it is shown. You see this old man is a controversial, you don't have to be like that. There is another person who is civilized, accepts a client well. And also the cleanliness we have learned. I got everything I got that day.

All intervention 3: Eee.The members of the household that, the customer should not miss him, you have to be a civilizator, whether you have a smile, show a smile, a customer loves you, but if you… it's true.

All intervention 3: The education I get is the way I get them and other things at home. there is one sister who… ..that a businessman is not supposed to be angry. Now I was trying to refer him to how we were taught here. That the master should be this, be this, be a client to the customer, be generous. … The client should not be missed, you must be a civilized person, that you have a smile, show a smile, a customer likes you

Learning Health related issues

Prevention and treatment of infectious diseases

Entrepreneurship 4: I am most comfortable with this health education program. Starting with epidemics and how to avoid it. Sexually transmitted diseases and how to avoid them. Health care how to go with it. Therefore, I am so comforted that I will be a teacher even in my family, that in everyday things especially health care services

And the second thing I have learned is the whole issue of health. I mean sexually transmitted diseases and other things

Health A 7: That is, in short, this project has been a leaven, a success in the overall life we live in. Starting from cleanliness, that is, in purity, until the pursuit of money. Due to the life we live in, It was before joining this project. It was very difficult when you worked there, then when it was time to eat it, that is, you eat with a spoon, but the hand can't wash, because you eat with a spoon. So there was a constant frequency of intestinal fever. But after getting a little health education, it was now even if you eat a teaspoon, You must have hands again with soap. In order for you to not get …… .In short, there has been… .and it has helped us with so many things, including our surroundings.

Health A 2: We were interested ... I was interested in health. I remember we learned about health. before we eat we have hands. When we leave the room we have hands. Protecting health We were taught about diarrhea

Beekeeping 8: Another time I learned, how to protect yourself from sexually transmitted diseases. Yes (Yes) .I have learned that you don't know us young people again! Young people have so many things, so there are other things we are going through, which we have to learn. So I have learned how to protect myself.

Health B 6: If you are talking to some of the worms, drinking hot water, filtering water, I did not know, I was just picking up a tap, drinking it. The master in the project flani, we have gone to learn what flies and flies, which you didn't know, I could tell you when you understand.

Entrepreneurship 5: First I knew that, if we missed oral, we can take one liter of water, we mix sugar and salt. we mixed up we got oral local. For this I also thank you for the health side I have also learned.

Importance of health education

Entrepreneurship 4: I was a hundred percent(100%) very comfortable with these health educations. Because health education cannot be found in nature. But these businesses, entrepreneurship is just sitting in Rombo who has been doing good business without education. But despite having a lot of money, you can't have health education. So you can have a lot of money, spend on health because of lack of education. So for a short time here I have learned all kinds of infections. From malaria, diarrhea, worms. We have been taught how to eat protein, nutrition, vitamins. We have been taught these first aid services.

Health A 2: That is like health education, because it was there every year, our illnesses, often. That is, in most cases, was widely used in hospitals. Because we had no education. After being taught, so far, we are grateful to God, our suffering has been gone.

Beekeeping 5: They taught us that human beings are, how to live, ie (yes), to avoid sexually transmitted diseases, if perhaps diseases or malaria, or in worms. And so many people, for ^. what I liked that perhaps the teacher by teaching us the issues of malaria, that, he taught us so many important things that are known but people are despising them, yes (yes) so.

Health 10: I have changed in two years since I get this education, it's knowing the symptoms. It is not a drink… ha, sir, I am sick… you vomit a little ah, I am suffering from malaria, or what I am suffering from. Spreading the symptoms, when I drink something, is not malaria, but is a symptom of worms, or something. I like that. And I know about it.

All intervention 7: All my friends know me. I liked sex very much -I was, pretty good. Yes, but there is sex education, here and what. Carefully comes in. it was changing me without knowing it. I was a condom to me was like …. you can't eat bananas and pods, you don't know those… this our street words eh. But I am grateful, I am here, my character right now is understandable .

First Aid

Entrepreneurship 5: Yes (yes), this has helped that even in a village where there are no health facilities, then I would be able to provide first aid, to someone who is suffering from stomach, or long-term vomiting.

Entrepreneurship 4: ……… but if these first aid services, you see eh, because we were compressed, that in such a person burning a fire), make honey, lay eggs, but it is not true. cool for twenty minutes (20).

Balanced diet

Beekeeping 8: I have loved something that is called nutrition. Nutrition I have learned how to eat…. these foods that we eat. Because there are other foods you can eat, and you find a disease you get as diabetes. So, I have learned what food I eat, that, you can eat what things become balanced diet.

Beekeeping 5: But right now, that I have benefited or changed, that, in the matter of…. I had the knowledge of the issues of this nutrition. So, I was for instance if I was eating unhealthy foods simply ie yes. Now at the end of the day I find myself eating and I am also the same as ah. Now the end of the day come to get here education - of about the dietary issues, the balanced diet, this and so on. My body is that there are foods that I have learned that if you eat this food and this and that, there is a body building, there is something to strengthen the body.

Beekeeping 5: Ya (yes), you have changed, and I know now that if I prepare this food and this, I can build a body, here I can strengthen the body, or give the body a strong body with this food.
